# The relationship between thyroid autoantibodies and X chromosome monosomy in the chorionic tissue of patients with missed miscarriage

**DOI:** 10.1186/s12958-024-01245-3

**Published:** 2024-06-20

**Authors:** Lu Zhao, Li Liu, Hua Yang

**Affiliations:** 1 Family Planning Department, Tianjin Central Hospital of Gynecology Obstetrics, Tianjin, 300010 China; 2 Ultrasound Department, Tianjin Central Hospital of Gynecology Obstetrics, Tianjin, 300010 China

**Keywords:** Missed early miscarriage, TGAb, TPOAb, Chorionic tissue chromosome

## Abstract

**Objective:**

The aim of this study was to investigate the relationship between thyroid autoantibodies (TGAb and TPOAb) and X chromosome monosomy in the chorionic tissue of patients with missed early miscarriage.

**Methods:**

The baseline data, thyroid function, thyroid antibody and the chromosomes from the chorionic tissue of 228 patients with missed early miscarriage were examined.

**Results:**

(1) Among the 228 patients, 121 had a normal chromosome number, and 107 had an abnormal chromosome number. The majority of them were autosomal trisomy, of which trisomy 16 (40.19%) was predominant. Sex chromosome monosomy (28.04%) was secondary. (2) Among the 228 patients, 208 patients in this study had normal thyroid function (including 134 cases of negative thyroid antibodies and 74 cases of positive thyroid antibodies alone); 6 patients had abnormal thyroid function (including 2 cases of clinical hyperthyroidism, 3 cases of subclinical hypothyroidism, 1 case of hypothyroxinemia); and 14 patients had normal TSH and elevated T4 alone.(3) After exclusion of patients with thyroid function abnormalities, there were no significant differences in baseline data between the normal chromosome group and the abnormal chromosome group (*P* > 0.05). However, there was a significant difference in TGAb and TPOAb between the normal chromosome and abnormal chromosome group with 45, X karyotype, with a higher proportion of TGAb and/or TPOAb positivity in the 45, X karyotype group (*P* < 0.05). Additionally, compared to TGAb and/or TPOAb-positive patients, the risk of X chromosome monosomy was significantly reduced in TGAb and TPOAb-negative patients (*P* < 0.05). Moreover, both TGAb and TPOAb titer values in the X chromosome monosomy group were higher than those in the chromosomally normal group (*P* < 0.05).

**Conclusion:**

There is a correlation between TGAb, TPOAb and X chromosome monosomy in the chorionic tissue of patients with missed early miscarriage, although the mechanism remains to be further investigated.

Thyroid autoantibodies in the human body, including thyroid peroxidase antibodies (TPOAb), thyroglobulin antibodies (TGAb), thyroid stimulating hormone receptor antibodies (TRAb), thyroid microsome antibodies (TMAb), and sodium/iodine symporter antibodies (NIS antibodies), can exist individually or simultaneously [1]. A positive thyroid antibody status during pregnancy is a special clinical condition among all the thyroid diseases during pregnancy. Whether it has adverse effects on both mother and baby has become a research hotspot in endocrinology, obstetrics and gynecology, and immunology in recent years. The thyroid antibodies of TPOAb and TGAb are thyroid autoimmune antibodies, which can accurately reflect the immune status of the thyroid. They are closely linked to adverse pregnancy outcomes, and are one of the primary detection items used in clinic to screen for adverse pregnancy factors. Currently, it is considered a multi-factor and multi-link complex process induced by various environmental factors and participated by various immune factors on the basis of genetic susceptibility [2, 3]. Missed early miscarriage refers to missed miscarriages occurring within ≤ 12 weeks of pregnancy, with an increasing incidence in recent years, reaching an annual morbidity rate of around 7% in singleton pregnancies [4]. Chromosomal abnormalities, especially abnormal chromosome number, are a primary cause of missed early miscarriage failure, with trisomy 16 and X chromosome monosomy being the most prevalent [5]. While TPOAb and TGAb are associated with missed early miscarriages, as well as environmental and genetic factors, there are currently no reports, both domestically and internationally, on the correlation between TGAb and TPOAb and abnormal chorionic tissue chromosome numbers in missed early miscarriages.

Therefore, this study aims to investigate the correlation between TGAb and TPOAb, and abnormal chorionic tissue chromosome numbers in missed early miscarriages, and it is expected to provide new approaches for the diagnosis and new targets for the treatment of this disorder.

## Materials and methods

### Main reagents and instruments

The Roche fully automatic electrochemiluminescence immunoassay system (purchased from Roche, Switzerland); the ABI 500 genetic sequencer (purchased from the Suzhou Genesky Biomedicine Company); the Free thyroxine (FT4) assay kit, the Free tri-iodothyronine (FT3) assay kit, the Thyroid stimulating hormone (TSH) assay kit, the Thyroglobulin antibody assay kit, the Thyroid peroxidase antibody assay kit (purchased from Roche Diagnostics (Shanghai) Ltd.).

### Subjects

According to the literature, the incidence of singleton pregnancy missed miscarriages is 7%. The PASS 15.0.1 software was used to calculate the sample size, with a significance level of α = 0.05 and an allowable error of 0.05. Based on the calculations, a sample size of 101 cases was required. Considering a dropout rate of 10%, no less than 111 cases were needed. A total of 228 women who experienced missed early miscarriages within ≤ 12 weeks of pregnancy were collected from September 2019 to December 2023 at the Tianjin Central Hospital of Gynecology Obstetrics. (a) Inclusion criteria: Women aged 20 to 35 with singleton pregnancies. (b) Exclusion criteria: Abnormal semen of the spouse, special dietary habits (strong tea, coffee, alcohol consumption, etc.), history of smoking, exposure to chemicals or radioactive substances, chronic diseases (such as hypertension, heart disease, diabetes, etc.), immune diseases(such as lupus erythematosus, antiphospholipid syndrome, sicca syndrome, etc.), hormonal abnormalities, abnormal coagulation function, reproductive system malformations, tumors, infections, etc.

Indicators and Detection Methods.

#### Determination of serum thyroid stimulating hormone (TSH), free thyroxine (FT4), thyroid peroxidase antibody (TPOAb), and thyroglobulin antibody (TGAb) in pregnant women

The researchers uniformly assigned numbers, punctured the median cubital vein from the arm of the research subjects, collected 5 ml of venous blood, allowed it to stand undisturbed at room temperature for 30 min, centrifuged it for 10 min in a low-speed centrifuge, extracted the upper serum layer, and dripped it into a 1.5mL polytetrafluoroethylene (PTFE) EP tube for detection (using the Roche fully automatic electrochemiluminescence immunoassay system).

#### Chorionic tissue chromosome testing

After the evacuation of the uterus, chorionic tissue was obtained, washed with sterile physiological saline, placed in a test tube, and cryopreserved throughout the entire process. The Multiplex Ligation-dependent Probe Amplification (MLPA) analysis technology was used to detect chorionic tissue chromosomes. Simultaneously, 2–3 ml of maternal venous blood was taken and sent as a reference to exclude whether the chorionic tissue chromosomes were affected by maternal contamination, thereby affecting the test results. 5 µL of DNA was processed at 98 °C for 5 min, cooled to room temperature, and then 3 µL of probe mixture was added. It was subjected to a 95 °C reaction for 1 min, followed by a 60 °C incubation for 16–24 h. The temperature of the PCR instrument was reduced to 54 °C, the tube cover was opened, and 32 µL of ligation enzyme mixture was added. After incubation at 54 °C, it was heated to 98 °C for 5 min to deactivate the ligation enzyme. The PCR instrument was then cooled to room temperature, 10 µL of PCR mixture was added under that same temperature, and the PCR reaction was initiated. Capillary electrophoresis was performed on the PCR product, and the results were analyzed using Coffalyser NET.

The normal reference values for thyroid hormones during the first trimester of pregnancy (Table 1, Table 2) are the recommended values found in the Specific Reference Range of Thyroid Function during Pregnancy issued in 2019 [6].

**Table 1:** Normal reference range of thyroid function during first trimester of pregnancy

Thyroid detection indicators	Median and two-sided limits (P2.5 and P97.5)
TSH (µIU/mL)	1.5 (0.03–4.51)
FT4 (pmol/L)	15.3 (11.8–21.0)
FT3 (pmol/L)	4.6 (3.6–5.6)
TPOAb (IU/mL)	> 5.61 is positive
TGAb (IU/mL)	> 4.11 is positive

**Table 2:** Criteria for thyroid function during first trimester of pregnancy

Thyroid function	TSH (µIU/mL)	FT4 (pmol/L)	TPOAb (IU/mL)		TGAb (IU/mL)
Normal	0.03–4.51	11.8–21.0	0-5.61	0-4.11	
Clinical hyperthyroidism	> 4.51	< 11.8	With or without antibody positivity		
Subclinical hyperthyroidism	> 4.51	11.8–21.0			
Hypothyroxinemia	0.03–4.51	< 11.8			
Clinical hyperthyreosis	< 0.03	> 21.0			
Subclinical hyperthyreosis	< 0.03	11.8–21.0			
Just positive for antibody	Normal Normal		> 5.61	> 4.11	

#### Ultrasound diagnostic criteria for missed miscarriage [7]

Intrauterine gestation sac ≥ 25 mm without an embryo seen; Absence of a gestation sac in the uterine cavity, and no embryo bud or fetal heartbeat seen after two weeks; Crown-rump length ≥ 7 mm without a fetal heartbeat; Presence of a yolk sac in the uterine cavity with no fetal heartbeat observed after 11 days.

### Statistical analysis

Data processing and statistical analysis were conducted using the SPSS 26.0 statistical software package. Quantitative data were expressed as mean ± SD after passed normality and homogeneity of variance tests, and analyzed using t-test analysis. Non-normally distributed data were compared using non-parametric tests. The qualitative data (ratios) were compared using chi-square tests. Logistic regression analysis was employed to analyze the risk relationship between TGAb and/or TPOAb positivity and various types of chromosomal abnormalities. *P*<0.05 indicated significant differences.

## Results

### Karyotype with chromosomal numerical abnormalities

Among the 228 patients, 121 cases (53.07%) exhibited a normal chromosomal number, while 107 cases (46.93%) displayed chromosomal numerical abnormalities. The most common abnormalities (71.96%, 77/107) were trisomies of autosomes, with trisomy 16 (40.19%, 43/107) being the most prevalent. Following this, sex chromosome monosomy accounted for 28.04% of the abnormal karyotypes (30/107; Table 3).

**Table 3:** Types and cases of chromosomal abnormalities of embryonic chorionic tissues in patients with missed miscarriage

Type	Karyotype	Number of Cases	Proportion (%)
Sex Chromosome Monosomy	45, X	30 Cases	28.04%
Autosomal trisomy		77 Cases	71.96%
	Trisomy 2	5 Cases	4.67%
	Trisomy 13	4 Cases	3.74%
	Trisomy 16	43 Cases	40.19%
	Trisomy 4	2 Cases	1.87%
	Trisomy 12	3 Cases	2.80%
	Trisomy 15	6 Cases	5.61%
	Trisomy 22	5 Cases	4.67%
	Trisomy 21	7 Cases	6.54%
	Trisomy 17	1 Cases	0.93%
	Trisomy 7	1 Cases	0.93%

### Thyroid function

Among the 228 patients, 208 patients(91.23%) in this study had normal thyroid function (including 134 cases of negative thyroid antibodies and 74 cases of positive thyroid antibodies alone); 6 patients (2.63%)had abnormal thyroid function (including 2 cases of clinical hyperthyroidism, 3 cases of subclinical hypothyroidism, 1 case of hypothyroxinemia); and 14 patients(6.14%) had normal TSH and elevated T4 alone.

### Baseline data of the normal chromosome group and the abnormal chromosome group (Table 4)

After exclusion of patients with thyroid function abnormalities, the remaining 228 patients were divided into a normal chromosomal group (108 cases) and an abnormal chromosomal group (100 cases). There were no significant differences between the two groups regarding age, gestational weeks, BMI, age at first pregnancy, gravidity and parity, history of missed miscarriages, or history of induced abortion. (for gravidity, Z = -1.28, *P* = 0.02; for the history of parity, Z = -0.32, *P* = 0.75; for the history of missed miscarriages, Z = 0.91, *P* = 0.36; for the induced abortion history, Z = -0.19, *P* = 0.84).

**Table 4:** Baseline data of the normal chromosome group and the abnormal chromosome group

General conditions	Normal group (x̄ ± s)	Abnormal group (x̄ ± s)	t Value	*P* Value
Age (Years)	30.28 ± 4.57	30.96 ± 4.14	-0.31	0.76
Gestational weeks (weeks)	8.44 ± 1.04	8.49 ± 1.11	-1.13	0.26
BMI (kg/m^2^)	22.77 ± 3.51	22.78 ± 3.52	-0.62	0.53
Age at first pregnancy (years)	27.03 ± 3.25	27.06 ± 4.38	-0.06	0.95

### The correlation between TGAb and TPOAb with abnormal chorionic tissue chromosome numbers in missed early miscarriages (Table 5)

After exclusion of the patients with thyroid function abnormalities among the 228 cases, we compared patients positive for TGAb and/or TPOAb with those negative. No significant differences were found in TGAb and TPOAb between the normal chromosomal group and the abnormal chromosomal group (χ^2^ = 1.64, *P* = 0.20); similarly, no significant differences were observed in TGAb and TPOAb among patients with the chromosomal abnormality of trisomy 16, between the normal and abnormal chromosomal groups (χ^2^ = 1.49, *P* = 0.22). However, there was a significant difference in TGAb and TPOAb between the normal chromosome group and the 45, X abnormal chromosome group; the proportion of TGAb and/or TPOAb positive patients in the 45, X group was higher (χ^2^ = 11.17, *P* = 0.001). Finally, there was no significant difference in TGAb and TPOAb between the normal chromosome group and the abnormal chromosome group with other abnormal karyotypes (χ^2^ = 0.71, *P* = 0.40).

**Table 5:** The proportions of TGAb and TPOAb in the normal chromosome group and the abnormal chromosome group

Chromosome status (cases)	TGAb and/or TPOAb positive [Number (%)]	TGAb and TPOAb negative [Number (%)]
Normal chromosome (108 cases)	34 (31.48%)	74 (68.52%)
Abnormal chromosome (100 cases)	40 (40%)	60 (60%)
Trisomy 16	8 (21.05%)	30 (78.95%)
45, X (29 cases)	19 (65.52%)	10 (34.48%)
Others (33 cases)	13 (39.39%)	20 (60.61%)

### The risk relationship between TGAb and/or TPOAb positive and different types of chromosomal numerical abnormalities

Patients with negative TGAb and TPOAb had a significantly lower risk of X chromatids (OR = 0.025, *P* = 0.007), the difference had statistically significant.

Patients with negative TGAb and TPOAb had no significant lower risk of trisomy 16 and other chromosome numerical abnormalities (OR = 0.182, *P* = 0.278) (OR = 0.166, *P* = 0.223).

### Risk relationship between TGAb and TPOAb titers and different types of chromosomal numerical abnormalities (Table 6)

The difference in TPOAb titer value in the chromosomally normal group and the X chromosome monosomy group was statistically significant (Z = − 3.52, *P* = 0.00). The difference in TGAb titer value in the chromosomally normal group and the X chromosome monosomy group was statistically significant (Z = − 2.72, *P* = 0.01).

The difference in TPOAb titer value in the chromosomally normal group and the trisomy 16 group was not statistically significant (Z = − 0.24, *P* = 0.81). The difference in TGAb titer value in the chromosomally normal group and the trisomy 16 group was not statistically significant (Z = − 1.29, *P* = 0.19).

The difference in TPOAb titer value in the chromosomally normal group and the other chromosomal abnormalities group was not statistically significant (Z = − 0.05, *P* = 0.96). The difference in TGAb titer value in the chromosomally normal group and the other chromosomal abnormalities group was not statistically significant (Z = − 1.21, *P* = 0.23).

**Table 6:** Correlation of TGAb and TPOAb titer values with different types of numerical chromosomal abnormalities

Chromosome status	TGAb titers(IU/mL) (P2.5 and P97.5)	TPOAb titers (IU/mL) (P2.5 and P97.5)
Normal chromosome	2.02(1.14–6.11)	0.51(0.15–4.36)
Trisomy 16	1.56(1.02–3.45)	0.52(0.1–0.89)
45, X	10.7(1.47–11.99)	9(0.49–10.62)
Others	1.45(0.82-10)	0.67(0.1-9)

## Discussion

The etiology of missed early miscarriage remains unclear, although it is currently believed to be associated with factors such as endocrine, immune, genetic, unhealthy lifestyle habits, and environmental factors. Among these, genetic and immune factors are the main causes of missed early miscarriage. Nearly 50% of miscarriages during the first-trimester of pregnancy are due to chromosomal abnormalities, particularly chromosomal numerical abnormalities. Some studies indicate that chromosomal numerical abnormalities account for miscarriages in 23–61% of cases [8, 9]. In this study, we conducted chromosomal testing on the embryonic chorionic tissues from 228 cases of missed miscarriage patients, and revealed 107 cases with chromosomal numerical abnormalities. Trisomy of autosomes was the most common, with trisomy 16 being predominant, followed by X chromosome monosomy, which is consistent with the previous research [10].

Currently, in clinical practice, it is believed that thyroid antibody positivity accompanied by thyroid dysfunction during pregnancy is a major cause of missed early miscarriage, which requires drug treatment. However, for pregnant women who were just positive for TPOAb and TGAb without thyroid dysfunction, regular monitoring of thyroid function is sufficient, and intervention or treatment is unnecessary [11]. Yet, Wegiel M et al. found that the Turner syndrome, with a prevalence of 1/2000–2500 in females, is often due to partial or complete loss of the X chromosome. Additionally, Turner syndrome patients have an increased risk of developing autoimmune thyroid disorder (AITD) [12]. Furthermore, a study which compared 158 AITD patients with positive TPOAb and TGAb and 181 healthy individuals demonstrated a correlation between AITD and gene copy number abnormalities [13]. Elevated thyroid antibodies are closely associated with familial chromosomal abnormalities, and the positive rate of thyroid autoantibodies in the first-degree relatives of AITD patients is also significantly higher than that in the general population [14].

Our study revealed significant differences in TGAb and TPOAb between the group with normal chromosomes and the group with chromosomal abnormalities, especially in the X chromosome monosomy subgroup. The proportion of individuals positive for TGAb and/or TPOAb was higher in the 45, X group, and compared to TGAb and/or TPOAb positive patients, those negative for TGAb and TPOAb showed a significant decrease in the risk of X chromosome monosomy. Both TGAb and TPOAb titer values in the X chromosome monosomy group were higher than those in the chromosomally normal group. Therefore, we speculate a correlation between TGAb, TPOAb, and embryonic X chromosome loss. Hence, close attention and appropriate treatment are still necessary for individuals who are just positive for TPOAb and TGAb, and its mechanism needs further investigation.

In summary, our study was conducted on the chorionic tissues from patients with missed early miscarriage and investigated the relationship between TGAb, TPOAb, and chromosomal numerical abnormalities in the chorionic tissue. The results may provide new approaches for the diagnosis and new targets for the treatment of the disease, which are meaningful for the subsequent pregnancy and smooth delivery of patients with missed early miscarriage. However, there are also some limitations. Our study only involved the population of Tianjin, China, which may be a factor of bias, and the sample size was small. Therefore, further extensive studies with larger sample size and more diverse populations are needed to confirm these findings and delve deeper into their mechanisms.

## Data Availability

The authors confirm that the data supporting the findings of this study are available within the article.
